# Diaqua­bis­[5-(pyrazin-2-yl-κ*N* ^1^)-3-(pyridin-4-yl)-1*H*-1,2,4-triazol-1-ido-κ*N* ^1^]cobalt(II) methanol disolvate

**DOI:** 10.1107/S160053681201495X

**Published:** 2012-04-18

**Authors:** Yan Bi, Na Wu, Jing Chen

**Affiliations:** a College of Chemistry, Tianjin Key Laboratory of Structure and Performance for Functional Molecules, Tianjin Normal University, Tianjin 300387, People’s Republic of China

## Abstract

The Co^II^ ion in the title mononuclear compound, [Co(C_11_H_7_N_6_)_2_(H_2_O)_2_]·2CH_3_OH, is located on an inversion center and is six-coordinated in a distorted octa­hedral geometry defined by four N atoms from two deprotonated 5-(pyrazin-2-yl-κ*N*)-3-(pyridin-4-yl)-1*H*-1,2,4-triazol-1-ide (ppt) ligands and two water mol­ecules. In the crystal, the complex mol­ecules and lattice methanol mol­ecules are linked *via* O—H⋯N and O—H⋯O hydrogen bonds, generating a two-dimensional supra­molecular network parallel to (001). π–π inter­actions between the triazole and pyrazine rings and between the pyridine rings are present [centroid–centroid distances = 3.686 (3) and 3.929 (4) Å, respectively].

## Related literature

For coordination complexes based on N-involved polydentate ligands, see: Guo *et al.* (2010 ▶); Ha (2011 ▶); Sun *et al.* (2011 ▶); Tang *et al.* (2011 ▶); Yang *et al.* (2010 ▶). For related structures based on 5-(pyrazin-2-yl)-3-(pyridin-4-yl)-1*H*-1,2,4-triazole, see: Liu *et al.* (2009 ▶).
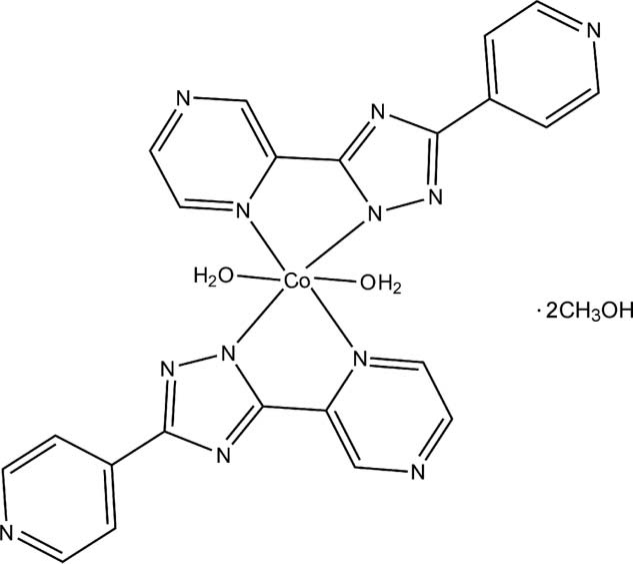


## Experimental

### 

#### Crystal data

[Co(C_11_H_7_N_6_)_2_(H_2_O)_2_]·2CH_4_O
*M* *_r_* = 605.50
Monoclinic, 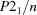

*a* = 11.462 (9) Å
*b* = 7.121 (5) Å
*c* = 16.116 (12) Å
β = 95.418 (14)°
*V* = 1309.6 (17) Å^3^
*Z* = 2
Mo *K*α radiation
μ = 0.71 mm^−1^
*T* = 296 K
0.36 × 0.22 × 0.10 mm

#### Data collection

Bruker APEX CCD diffractometer
Absorption correction: multi-scan (*SADABS*; Bruker, 2001 ▶) *T*_min_ = 0.783, *T*_max_ = 0.932
6377 measured reflections
2307 independent reflections
1685 reflections with *I* > 2σ(*I*)
*R*_int_ = 0.039

#### Refinement

*R*[*F* ^2^ > 2σ(*F* ^2^)] = 0.040
*wR*(*F* ^2^) = 0.104
*S* = 1.03
2307 reflections
189 parameters
H-atom parameters constrained
Δρ_max_ = 0.22 e Å^−3^
Δρ_min_ = −0.31 e Å^−3^

### 

Data collection: *SMART* (Bruker, 2007 ▶); cell refinement: *SAINT* (Bruker, 2007 ▶); data reduction: *SAINT*; program(s) used to solve structure: *SHELXS97* (Sheldrick, 2008 ▶); program(s) used to refine structure: *SHELXL97* (Sheldrick, 2008 ▶); molecular graphics: *DIAMOND* (Brandenburg, 1999 ▶); software used to prepare material for publication: *SHELXTL* (Sheldrick, 2008 ▶).

## Supplementary Material

Crystal structure: contains datablock(s) I, global. DOI: 10.1107/S160053681201495X/hy2533sup1.cif

Structure factors: contains datablock(s) I. DOI: 10.1107/S160053681201495X/hy2533Isup2.hkl

Additional supplementary materials:  crystallographic information; 3D view; checkCIF report

## Figures and Tables

**Table 1:** Hydrogen-bond geometry (Å, °)

*D*—H⋯*A*	*D*—H	H⋯*A*	*D*⋯*A*	*D*—H⋯*A*
O2—H2⋯N6^i^	0.82	1.97	2.760 (4)	163
O1—H1*B*⋯N5^ii^	0.85	1.94	2.785 (3)	176
O1—H1*A*⋯O2^iii^	0.85	1.81	2.660 (3)	173

Symmetry codes: (i) 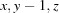; (ii) 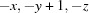; (iii) 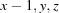.

**Fig. 1.**
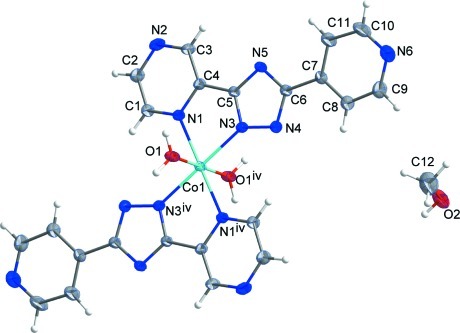
Molecular structure of the title complex, showing displacement ellipsoids drawn at the 30% probability level. [Symmetry code: (iv) -x, -y, -z.]

**Fig. 2.**
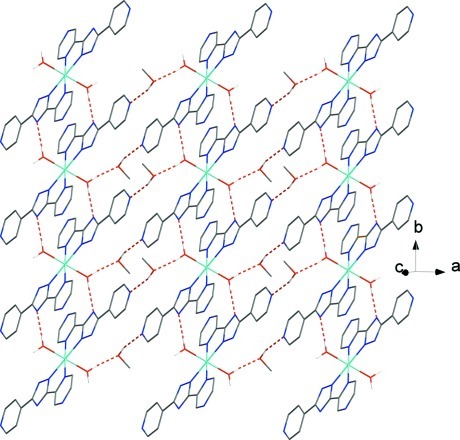
View of the two-dimensional supramolecular network linked via O—H···O and O—H···N hydrogen bonds (red dashed lines).

## supplementary crystallographic information

### Comment

The selection of organic ligands is generally considered as the critical factor for constructing metallosupramolecular complexes. In this connection, nitrogen-involved polydentate ligands have attracted special attentions because of their preference and reliability for coordinating to transition metal ions in versatile fashions (Guo *et al.*, 2010; Ha, 2011; Sun *et al.*, 2011; Tang *et al.*, 2011; Yang *et al.*, 2010). For example, 5-(pyrazin-2-yl)-3-(pyridin-4-yl)-1*H*-1,2,4-triazole (Hppt) has been recently used to prepare two Cu(II) complexes with the observation of unique structural transformations (Liu *et al.*, 2009). Herein, the reaction of Hppt with Co(NO_3_)_2_.6H_2_O produces the title mononuclear complex.

The asymmetric unit of the title complex consists of a Co^II^ ion that lies on an inversion center, one deprotonated ppt anion, one water ligand and one lattice methanol molecule. As shown in Fig. 1, the Co^II^ ion takes a distorted octahedral geometry, coordinating to four N atoms from two ppt ligands [Co—N = 2.076 (2) and 2.130 (2) Å] in the equatorial plane and to two axial water ligands [Co—O = 2.087 (2) Å]. The deprotonated ppt ligand adopts a chelating mode through both the pyrazinyl and triazolyl N donors.

As shown in Fig. 2, the lattice methanol molecule is bonded to the water ligand *via* O1—H1A···O2^iii^ and the uncoordinated pyridyl group of the ppt ligand *via* O2—H2···N6^i^ hydrogen bonds [symmetry codes: (i) *x*, -1+*y*, *z*; (iii) -1+*x*, *y*, *z*], linking the adjacent mononuclear complexes into a two-dimensional network. O1—H1B···N5^ii^ hydrogen bond [symmetry code: (ii) -*x*, 1-*y*, -*z*] between the coordinated water and triazole ring is also observed to reinforce this two-dimensional network. In addition, aromatic stacking interactions between the triazolyl (N3—N5, C5, C6) and pyrazinyl (N1, N2, C1—C4) rings as well as between the parallel pyridyl groups (N6, C7—C11) are also found within this supramolecular layer, with centroid–centroid distances and dihedral angles of 3.686 (3)/3.929 (4) Å and 4.2/0.0°.

### Experimental

A CH_3_OH solution (3 ml) of Hppt (11.2 mg, 0.05 mmol) was carefully layered onto an aqueous solution (5 ml) of Co(NO_3_)_2_.6H_2_O (29.1 mg, 0.1 mmol) in a straight glass tube. After evaporating the solvents slowly for *ca* 1 week, yellow block single crystals suitable for X-ray diffraction analysis were obtained in *ca* 40% yield. Analysis, calculated for C_24_H_26_CoN_12_O_4_: C 47.61, H 4.33, N 27.76%; found: C 48.02, H 4.19, N 27.89%.

### Refinement

All H atoms were initially located in a difference Fourier map, then constrained to an ideal geometry and refined as riding atoms, with C—H = 0.93 (aromatic) and 0.96 (methyl) Å and O—H = 0.85 (water) and 0.82 (methanol) Å and with *U*_iso_(H) = 1.2*U*_eq_(C) and 1.5*U*_eq_(O).

### Crystal data

**Table:**

[Co(C_11_H_7_N_6_)_2_(H_2_O)_2_]·2CH_4_O	*F*(000) = 626
*M**_r_* = 605.50	*D*_x_ = 1.536 Mg m^−^^3^
Monoclinic, *P*2_1_/*n*	Mo *K*α radiation, λ = 0.71073 Å
Hall symbol: -P 2yn	Cell parameters from 1325 reflections
*a* = 11.462 (9) Å	θ = 2.5–22.3°
*b* = 7.121 (5) Å	µ = 0.71 mm^−^^1^
*c* = 16.116 (12) Å	*T* = 296 K
β = 95.418 (14)°	Block, yellow
*V* = 1309.6 (17) Å^3^	0.36 × 0.22 × 0.10 mm
*Z* = 2	

### Data collection

**Table:**

Bruker APEX CCD diffractometer	2307 independent reflections
Radiation source: fine-focus sealed tube	1685 reflections with *I* > 2σ(*I*)
Graphite monochromator	*R*_int_ = 0.039
φ and ω scans	θ_max_ = 25.0°, θ_min_ = 2.1°
Absorption correction: multi-scan (*SADABS*; Bruker, 2001)	*h* = −13→13
*T*_min_ = 0.783, *T*_max_ = 0.932	*k* = −8→7
6377 measured reflections	*l* = −16→19

### Refinement

**Table:**

Refinement on *F*^2^	Primary atom site location: structure-invariant direct methods
Least-squares matrix: full	Secondary atom site location: difference Fourier map
*R*[*F*^2^ > 2σ(*F*^2^)] = 0.040	Hydrogen site location: inferred from neighbouring sites
*wR*(*F*^2^) = 0.104	H-atom parameters constrained
*S* = 1.03	*w* = 1/[σ^2^(*F*_o_^2^) + (0.0486*P*)^2^ + 0.3583*P*] where *P* = (*F*_o_^2^ + 2*F*_c_^2^)/3
2307 reflections	(Δ/σ)_max_ < 0.001
189 parameters	Δρ_max_ = 0.22 e Å^−^^3^
0 restraints	Δρ_min_ = −0.31 e Å^−^^3^

### Special details

**Table:**

Geometry. All e.s.d.'s (except the e.s.d. in the dihedral angle between two l.s. planes) are estimated using the full covariance matrix. The cell e.s.d.'s are taken into account individually in the estimation of e.s.d.'s in distances, angles and torsion angles; correlations between e.s.d.'s in cell parameters are only used when they are defined by crystal symmetry. An approximate (isotropic) treatment of cell e.s.d.'s is used for estimating e.s.d.'s involving l.s. planes.
Refinement. Refinement of *F*^2^ against ALL reflections. The weighted *R*-factor *wR* and goodness of fit *S* are based on *F*^2^, conventional *R*-factors *R* are based on *F*, with *F* set to zero for negative *F*^2^. The threshold expression of *F*^2^ > σ(*F*^2^) is used only for calculating *R*-factors(gt) *etc*. and is not relevant to the choice of reflections for refinement. *R*-factors based on *F*^2^ are statistically about twice as large as those based on *F*, and *R*- factors based on ALL data will be even larger.

### Fractional atomic coordinates and isotropic or equivalent isotropic displacement parameters (Å^2^)

**Table:**

	*x*	*y*	*z*	*U*_iso_*/*U*_eq_	
Co1	0.0000	0.0000	0.0000	0.03537 (19)	
O1	−0.09917 (17)	0.1407 (3)	0.08265 (12)	0.0466 (5)	
H1A	−0.1489	0.0789	0.1075	0.070*	
H1B	−0.1214	0.2496	0.0663	0.070*	
O2	0.7363 (2)	−0.0266 (4)	0.16227 (19)	0.0799 (8)	
H2	0.6906	−0.1150	0.1557	0.120*	
N1	−0.06613 (19)	0.1782 (3)	−0.09975 (14)	0.0354 (6)	
N2	−0.1322 (2)	0.4390 (4)	−0.22262 (17)	0.0549 (8)	
N3	0.1212 (2)	0.2174 (3)	0.00719 (14)	0.0375 (6)	
N4	0.2220 (2)	0.2651 (3)	0.05380 (15)	0.0414 (6)	
N5	0.1735 (2)	0.4979 (3)	−0.03782 (14)	0.0364 (5)	
N6	0.5474 (3)	0.7349 (5)	0.1316 (2)	0.0752 (10)	
C1	−0.1573 (3)	0.1507 (4)	−0.15428 (18)	0.0438 (8)	
H1	−0.2012	0.0415	−0.1512	0.053*	
C2	−0.1893 (3)	0.2791 (4)	−0.2156 (2)	0.0527 (9)	
H2A	−0.2535	0.2530	−0.2536	0.063*	
C3	−0.0408 (3)	0.4681 (4)	−0.16709 (19)	0.0454 (8)	
H3	0.0009	0.5797	−0.1693	0.055*	
C4	−0.0058 (2)	0.3391 (4)	−0.10655 (17)	0.0353 (7)	
C5	0.0967 (2)	0.3570 (4)	−0.04610 (17)	0.0333 (7)	
C6	0.2498 (2)	0.4331 (4)	0.02493 (18)	0.0371 (7)	
C7	0.3528 (3)	0.5369 (4)	0.06056 (19)	0.0422 (8)	
C8	0.4329 (3)	0.4564 (5)	0.1186 (2)	0.0639 (10)	
H8	0.4235	0.3328	0.1354	0.077*	
C9	0.5269 (3)	0.5597 (6)	0.1513 (3)	0.0807 (13)	
H9	0.5801	0.5018	0.1903	0.097*	
C10	0.4694 (4)	0.8116 (6)	0.0775 (3)	0.0824 (13)	
H10	0.4802	0.9367	0.0636	0.099*	
C11	0.3724 (3)	0.7207 (5)	0.0397 (2)	0.0663 (11)	
H11	0.3211	0.7826	0.0008	0.080*	
C12	0.6909 (4)	0.1064 (7)	0.2107 (3)	0.1041 (16)	
H12A	0.6849	0.0563	0.2654	0.156*	
H12B	0.6145	0.1422	0.1863	0.156*	
H12C	0.7413	0.2143	0.2145	0.156*	

### Atomic displacement parameters (Å^2^)

**Table:**

	*U*^11^	*U*^22^	*U*^33^	*U*^12^	*U*^13^	*U*^23^
Co1	0.0389 (3)	0.0217 (3)	0.0427 (3)	−0.0062 (2)	−0.0105 (2)	0.0049 (2)
O1	0.0551 (13)	0.0266 (11)	0.0577 (14)	−0.0033 (9)	0.0037 (11)	0.0067 (9)
O2	0.0714 (18)	0.0657 (18)	0.104 (2)	−0.0281 (14)	0.0175 (17)	−0.0126 (16)
N1	0.0378 (13)	0.0258 (12)	0.0405 (14)	−0.0034 (11)	−0.0076 (11)	0.0018 (10)
N2	0.0617 (18)	0.0399 (15)	0.0577 (18)	−0.0012 (13)	−0.0231 (15)	0.0110 (13)
N3	0.0373 (13)	0.0268 (12)	0.0457 (14)	−0.0045 (10)	−0.0106 (11)	0.0061 (11)
N4	0.0371 (13)	0.0325 (13)	0.0516 (15)	−0.0061 (11)	−0.0122 (12)	0.0037 (12)
N5	0.0380 (13)	0.0237 (12)	0.0460 (14)	−0.0063 (11)	−0.0038 (11)	0.0025 (11)
N6	0.060 (2)	0.064 (2)	0.096 (2)	−0.0251 (17)	−0.0236 (18)	0.0072 (19)
C1	0.0445 (17)	0.0324 (16)	0.0510 (19)	−0.0083 (14)	−0.0149 (15)	0.0006 (14)
C2	0.054 (2)	0.0427 (19)	0.057 (2)	−0.0076 (16)	−0.0208 (17)	0.0035 (16)
C3	0.0520 (19)	0.0308 (17)	0.0499 (19)	−0.0047 (14)	−0.0138 (16)	0.0088 (14)
C4	0.0387 (16)	0.0270 (14)	0.0385 (17)	0.0004 (12)	−0.0058 (13)	0.0007 (13)
C5	0.0351 (15)	0.0249 (14)	0.0383 (17)	−0.0041 (12)	−0.0053 (13)	0.0008 (12)
C6	0.0376 (16)	0.0281 (14)	0.0437 (18)	−0.0043 (12)	−0.0052 (14)	0.0010 (13)
C7	0.0377 (17)	0.0366 (18)	0.0510 (19)	−0.0062 (13)	−0.0030 (14)	−0.0005 (14)
C8	0.050 (2)	0.049 (2)	0.087 (3)	−0.0112 (16)	−0.021 (2)	0.0122 (19)
C9	0.056 (2)	0.064 (3)	0.113 (3)	−0.0139 (19)	−0.038 (2)	0.009 (2)
C10	0.086 (3)	0.060 (3)	0.095 (3)	−0.040 (2)	−0.022 (3)	0.020 (2)
C11	0.065 (2)	0.052 (2)	0.076 (2)	−0.0246 (18)	−0.024 (2)	0.0138 (19)
C12	0.085 (3)	0.096 (4)	0.134 (4)	−0.010 (3)	0.023 (3)	−0.029 (3)

### Geometric parameters (Å, º)

**Table:**

Co1—N3^i^	2.076 (2)	N6—C9	1.314 (5)
Co1—N3	2.076 (2)	C1—C2	1.370 (4)
Co1—O1^i^	2.087 (2)	C1—H1	0.9300
Co1—O1	2.087 (2)	C2—H2A	0.9300
Co1—N1^i^	2.130 (2)	C3—C4	1.372 (4)
Co1—N1	2.130 (2)	C3—H3	0.9300
O1—H1A	0.8502	C4—C5	1.460 (4)
O1—H1B	0.8501	C6—C7	1.464 (4)
O2—C12	1.361 (5)	C7—C8	1.373 (4)
O2—H2	0.8200	C7—C11	1.375 (4)
N1—C1	1.315 (3)	C8—C9	1.369 (5)
N1—C4	1.348 (3)	C8—H8	0.9300
N2—C2	1.324 (4)	C9—H9	0.9300
N2—C3	1.328 (4)	C10—C11	1.378 (5)
N3—C5	1.326 (3)	C10—H10	0.9300
N3—N4	1.361 (3)	C11—H11	0.9300
N4—C6	1.333 (4)	C12—H12A	0.9600
N5—C5	1.332 (3)	C12—H12B	0.9600
N5—C6	1.354 (3)	C12—H12C	0.9600
N6—C10	1.307 (5)		
			
N3^i^—Co1—N3	180.00 (12)	C1—C2—H2A	118.8
N3^i^—Co1—O1^i^	90.47 (10)	N2—C3—C4	122.3 (3)
N3—Co1—O1^i^	89.53 (10)	N2—C3—H3	118.9
N3^i^—Co1—O1	89.53 (10)	C4—C3—H3	118.9
N3—Co1—O1	90.47 (10)	N1—C4—C3	120.6 (3)
O1^i^—Co1—O1	180.00 (14)	N1—C4—C5	114.0 (2)
N3^i^—Co1—N1^i^	77.67 (9)	C3—C4—C5	125.3 (3)
N3—Co1—N1^i^	102.33 (9)	N3—C5—N5	113.7 (2)
O1^i^—Co1—N1^i^	91.11 (10)	N3—C5—C4	118.3 (2)
O1—Co1—N1^i^	88.89 (10)	N5—C5—C4	128.0 (2)
N3^i^—Co1—N1	102.33 (9)	N4—C6—N5	114.1 (2)
N3—Co1—N1	77.67 (9)	N4—C6—C7	121.8 (3)
O1^i^—Co1—N1	88.89 (10)	N5—C6—C7	124.2 (2)
O1—Co1—N1	91.11 (10)	C8—C7—C11	116.7 (3)
N1^i^—Co1—N1	180.00 (17)	C8—C7—C6	121.3 (3)
Co1—O1—H1A	118.9	C11—C7—C6	122.0 (3)
Co1—O1—H1B	113.9	C9—C8—C7	119.3 (3)
H1A—O1—H1B	115.1	C9—C8—H8	120.4
C12—O2—H2	109.5	C7—C8—H8	120.4
C1—N1—C4	117.0 (2)	N6—C9—C8	124.7 (4)
C1—N1—Co1	128.27 (19)	N6—C9—H9	117.6
C4—N1—Co1	114.77 (18)	C8—C9—H9	117.6
C2—N2—C3	116.2 (3)	N6—C10—C11	124.8 (4)
C5—N3—N4	106.7 (2)	N6—C10—H10	117.6
C5—N3—Co1	115.07 (17)	C11—C10—H10	117.6
N4—N3—Co1	138.23 (18)	C7—C11—C10	118.9 (3)
C6—N4—N3	104.4 (2)	C7—C11—H11	120.6
C5—N5—C6	101.1 (2)	C10—C11—H11	120.6
C10—N6—C9	115.5 (3)	O2—C12—H12A	109.5
N1—C1—C2	121.6 (3)	O2—C12—H12B	109.5
N1—C1—H1	119.2	H12A—C12—H12B	109.5
C2—C1—H1	119.2	O2—C12—H12C	109.5
N2—C2—C1	122.3 (3)	H12A—C12—H12C	109.5
N2—C2—H2A	118.8	H12B—C12—H12C	109.5
			
N3^i^—Co1—N1—C1	−3.1 (3)	N2—C3—C4—C5	176.9 (3)
N3—Co1—N1—C1	176.9 (3)	N4—N3—C5—N5	−1.0 (3)
O1^i^—Co1—N1—C1	87.1 (3)	Co1—N3—C5—N5	177.47 (18)
O1—Co1—N1—C1	−92.9 (3)	N4—N3—C5—C4	177.7 (2)
N3^i^—Co1—N1—C4	176.62 (19)	Co1—N3—C5—C4	−3.9 (3)
N3—Co1—N1—C4	−3.38 (19)	C6—N5—C5—N3	0.8 (3)
O1^i^—Co1—N1—C4	−93.1 (2)	C6—N5—C5—C4	−177.7 (3)
O1—Co1—N1—C4	86.9 (2)	N1—C4—C5—N3	0.9 (4)
O1^i^—Co1—N3—C5	92.8 (2)	C3—C4—C5—N3	−178.0 (3)
O1—Co1—N3—C5	−87.2 (2)	N1—C4—C5—N5	179.4 (3)
N1^i^—Co1—N3—C5	−176.2 (2)	C3—C4—C5—N5	0.4 (5)
N1—Co1—N3—C5	3.8 (2)	N3—N4—C6—N5	−0.2 (3)
O1^i^—Co1—N3—N4	−89.4 (3)	N3—N4—C6—C7	178.5 (3)
O1—Co1—N3—N4	90.6 (3)	C5—N5—C6—N4	−0.4 (3)
N1^i^—Co1—N3—N4	1.7 (3)	C5—N5—C6—C7	−179.0 (3)
N1—Co1—N3—N4	−178.3 (3)	N4—C6—C7—C8	7.8 (5)
C5—N3—N4—C6	0.7 (3)	N5—C6—C7—C8	−173.7 (3)
Co1—N3—N4—C6	−177.3 (2)	N4—C6—C7—C11	−170.2 (3)
C4—N1—C1—C2	0.4 (4)	N5—C6—C7—C11	8.3 (5)
Co1—N1—C1—C2	−179.9 (2)	C11—C7—C8—C9	−0.8 (6)
C3—N2—C2—C1	0.6 (5)	C6—C7—C8—C9	−178.9 (4)
N1—C1—C2—N2	−1.3 (5)	C10—N6—C9—C8	1.1 (7)
C2—N2—C3—C4	1.1 (5)	C7—C8—C9—N6	0.2 (7)
C1—N1—C4—C3	1.2 (4)	C9—N6—C10—C11	−1.9 (7)
Co1—N1—C4—C3	−178.6 (2)	C8—C7—C11—C10	0.0 (6)
C1—N1—C4—C5	−177.8 (3)	C6—C7—C11—C10	178.1 (3)
Co1—N1—C4—C5	2.4 (3)	N6—C10—C11—C7	1.5 (7)
N2—C3—C4—N1	−2.0 (5)		

Symmetry code: (i) −*x*, −*y*, −*z*.

### Hydrogen-bond geometry (Å, º)

**Table:**

*D*—H···*A*	*D*—H	H···*A*	*D*···*A*	*D*—H···*A*
O2—H2···N6^ii^	0.82	1.97	2.760 (4)	163
O1—H1*B*···N5^iii^	0.85	1.94	2.785 (3)	176
O1—H1*A*···O2^iv^	0.85	1.81	2.660 (3)	173

Symmetry codes: (ii) *x*, *y*−1, *z*; (iii) −*x*, −*y*+1, −*z*; (iv) *x*−1, *y*, *z*.
